# A standard procedure for creating a frailty index

**DOI:** 10.1186/1471-2318-8-24

**Published:** 2008-09-30

**Authors:** Samuel D Searle, Arnold Mitnitski, Evelyne A Gahbauer, Thomas M Gill, Kenneth Rockwood

**Affiliations:** 1 Geriatric Medicine Research Unit, Dalhousie University & Capital District Health Authority, Halifax, Canada; 2 Department of Medicine, Dalhousie University, Halifax, Canada; 3 Department of Mathematics & Statistics, Dalhousie University, Halifax, Canada; 4 Department of Internal Medicine, Yale University School of Medicine, New Haven, CT 06504, USA; 5 Division of Geriatric Medicine, Dalhousie University, Halifax, Canada

## Abstract

**Background:**

Frailty can be measured in relation to the accumulation of deficits using a frailty index. A frailty index can be developed from most ageing databases. Our objective is to systematically describe a standard procedure for constructing a frailty index.

**Methods:**

This is a secondary analysis of the Yale Precipitating Events Project cohort study, based in New Haven CT. Non-disabled people aged 70 years or older (n = 754) were enrolled and re-contacted every 18 months. The database includes variables on function, cognition, co-morbidity, health attitudes and practices and physical performance measures. Data came from the baseline cohort and those available at the first 18-month follow-up assessment.

**Results:**

Procedures for selecting health variables as candidate deficits were applied to yield 40 deficits. Recoding procedures were applied for categorical, ordinal and interval variables such that they could be mapped to the interval 0–1, where 0 = absence of a deficit, and 1= full expression of the deficit. These individual deficit scores were combined in an index, where 0= no deficit present, and 1= all 40 deficits present. The values of the index were well fit by a gamma distribution. Between the baseline and follow-up cohorts, the age-related slope of deficit accumulation increased from 0.020 (95% confidence interval, 0.014–0.026) to 0.026 (0.020–0.032). The 99% limit to deficit accumulation was 0.6 in the baseline cohort and 0.7 in the follow-up cohort. Multivariate Cox analysis showed the frailty index, age and sex to be significant predictors of mortality.

**Conclusion:**

A systematic process for creating a frailty index, which relates deficit accumulation to the individual risk of death, showed reproducible properties in the Yale Precipitating Events Project cohort study. This method of quantifying frailty can aid our understanding of frailty-related health characteristics in older adults.

## Background

Frailty is a state of increased vulnerability to adverse outcomes. How best to operationalize frailty is controversial [1-3], but one method uses a frailty index [4]. The principle is to count *deficits in health *(which can be symptoms, signs, diseases, disabilities or laboratory, radiographic or electrocardiographic abnormalities) on the grounds that the more deficits a person has, the more likely that person is to be frail. The index is often expressed as a ratio of deficits present to the total number of deficits considered. For example, if 40 deficits were considered, and 10 were present in a given person, that person's frailty index would be 10/40 = 0.25.

Although the idea and approach are relatively simple, the results yielded by the frailty index have been consistent between surveys evaluated by our group [4-7] and by others [8-11] even though not every frailty index considers the same deficits, or even the same number of deficits. For example, across several frailty index measures, people accumulate deficits, on average, at about 0.03/year [4,5]. In each study, the frailer the person is (the higher the deficit count) the more vulnerable they are to adverse outcomes [5,8,11]. The frailty index is strongly associated with the risk of death, institutionalization and worsening health status, especially when at least 30 variables are included [5]. The frailty index also shows a consistent, sub-maximal limit at about 2/3 of the deficits that are considered. For example, if a frailty index is composed of 60 items, the most that anyone will have wrong with them is not 60, but 40 [5].

The reproducibility of the findings in relation to the frailty index is of some interest because none of the samples in which the frailty index has been operationalized has considered the same deficits. To be clear, it does not matter if study A considered 40 deficits from set X of deficits and study B considered 60 deficits from set Y of deficits; the estimates from each (e.g. the rate of deficit accumulation, the relationship between deficit accumulation and mortality, or the limit to deficit accumulation) appear to be similar. This finding suggests that frailty is a real phenomenon, which is a property of a biologically complex system. It indicates that frailty can be measured in many ways, and therefore can be studied in many existing datasets that might not have set out to measure frailty per se. To encourage more widespread evaluation of frailty – a goal encouraged by many groups [12-14] – we present a detailed, step-by-step procedure to describe which potential variables can be included in a frailty index, and how to establish cut-points for continuous variables. Here, frailty indexes were newly created using baseline and follow-up samples from an existing cohort study, and their properties (e.g. rate of increase, limit, and relationship with mortality) were compared with each other, and with earlier work.

## Methods

### The Study Sample

The Yale Precipitating Events Project (PEP) is a cohort study based in New Haven CT that enrolled individuals aged 70 years or older. Its methods have been published elsewhere [15,16]. Briefly, 754 community dwelling, English speaking, non-disabled persons with life expectancy and plans to stay in the area for more than 12 months were enrolled in the study. Comprehensive home-based assessments were completed at baseline and every 18 months. The 18-month assessment included 681 participants aged 72 to 98 years. This report uses the baseline and 18 month follow up data to contrast with each other and to compare the properties of the new indexes created in this data set with previously reported frailty indexes. At baseline, most participants (n = 487, 64.6%) were women, and most (n = 682, 90.5%) were white, with a mean Mini-Mental State Examination (MMSE) [17] score of 26.8 (SD = 2.50). Mortality was checked monthly for nine years from the baseline interview and was confirmed by obituaries and death certificates.

### Selecting candidate deficits for the Frailty Index

A frailty index counts *deficits in health*. These deficits were defined as symptoms, signs, disabilities and diseases [5]. All health deficits, including continuous, ordinal and binary variables, were taken from the PEP survey data dictionary. Restricted activity, disability in Activities Daily Living (ADL) and Instrumental ADL, impairments in general cognition and physical performance (e.g. impaired grip strength, impaired walking), co-morbidity, self-rated health, and depression/mood were evaluated.

Variables can be included in a frailty index if they satisfy the following 5 criteria:

1) The variables must be deficits associated with health status. Attributes such as graying hair, while age-related, are attributes and therefore not included. 2) A deficit's prevalence must generally increase with age, although some clearly age-related adverse conditions can decrease in prevalence at very advanced ages due to survivor effects. 3) Similarly, the chosen deficits must not saturate too early. For instance, age-related lens changes resulting in problems with accommodation (presbyopia) are nearly universal by age 55; in other words, as a variable, presbyopia saturates too early to be considered as a deficit here. 4) When considering the candidate deficits as a group, the deficits that make up a frailty index must cover a range of systems – if all variables were related to cognition, for example, the resulting index might well describe changes in cognition over time, but would be a cognitive impairment index [18] not a frailty index. 5) If a single frailty index is to be used serially on the same people, the items that make up the frailty index need to be the same from one iteration to the next [19]. The requirement to use the same items need not apply to comparisons between samples – i.e. samples that use difference frailty indexes appear to yield similar results [5].

Deficits should be added until there are at least 30–40 total deficits. There needs to be a minimum number of deficits. In general, the more variables that are included in a frailty index, the more precise estimates become. Similarly, estimates are unstable when the number of deficits is small – about 10 or less. Even so, an index with 30–40 variables has been shown to be sufficiently accurate for predicting adverse outcomes [6,14]. Furthermore, a frailty index can be constructed using information that is readily available in most health surveys, and is clinically tractable – i.e. it uses an amount that would be gathered in many routine health assessments of older adults [5].

### Coding of individual variables

All binary variables were recoded, using the convention that '0' indicated the absence of the deficit, and '1' the presence of a deficit. For variables that included a single intermediate response (e.g. 'sometimes' or 'maybe'), we used an additional value of '0.5'.

Frailty index variables can also accommodate ordinal and continuous variables as deficits. To do so requires grading the continuum or rank into a score between 0 (where no deficit is present) and 1 (where the deficit is maximally expressed by the given variable). For some variables, this re-coding is self-evident. Consider the widely used Self-rated Health Question ("How would you rate your health? Excellent, Very Good, Good, Fair, Poor"). To grade this between '0' and '1', each lower self-rating of health was coded to represent a larger deficit ("Excellent = 0", "Very Good = 0.25", "Good = 0.5", "Fair = 0.75" and "Poor = 1"). Similarly, recognized cut-points can be used for ordinal and continuous variables, such as the rapid walk test [15]. For the MMSE, we recoded deficits according to severity of impairment [20]. We assigned a 1 for scores less than 10, denoting severe dementia, 0.75 for scores ≥ 10 and ≤ 17, denoting moderate dementia, 0.5 for scores ≥ 18 and ≤ 20, denoting mild dementia, 0.25 for scores >20 and <24, denoting mild cognitive impairment (MCI), and 0 for scores ≥ 24, denoting no cognitive impairment [20]. Some readers might object that a score of '1' seems something of a discount (not a sufficiently high count) for severe dementia, and that losing only 1 point for it, compared with 0.25 points for MCI is not valid on its face. Consider, however, that a person with severe dementia is likely to have many more deficits than a person with MCI, e.g. more disability, poorer physical performance, higher degrees of behavioural problems and so forth.

Because not all ordinal or continuous variables have published or self-evident cut-points, additional work is required to establish the least arbitrary cut-points for these variables. Methods to address this can be broadly categorized as those based on characteristics of the distribution and those based on judgment (e.g. in relation to some clinically relevant hazard) [21]. Here, we employ both approaches. We used all existing previously coded deficits to establish an interim frailty index, whose purpose was to help provide cut-points for the remaining variables. This interim/nearly completed index was then plotted against the remaining ordinal and continuous variables to understand where their cut-points might be determined. The value of the individual variable that corresponded to 0.2 on the interim frailty index, i.e. the value of the variable at which, on average people had a frailty score of 0.2 or higher, was denoted as that deficit's cut-point. The value 0.2 on the frailty index is recognized by multiple frailty measures as approaching a frail state [7,8,22], so that this method met the convention of defining deficit cut-points. In addition, setting the value at, say, 0.3 seems unreasonably high, as this is consistently well into the range of frailty, however, defined (including by an increased hazard) so would be insensitive. Greater sensitivity is obtained at a cut-point of 0.1, but with less specificity.

### Analysis of Baseline and Follow up Cohorts

The rate of accumulation of deficits was calculated by evaluating the slope of a best fit log of the frailty index in relation to age. To evaluate the impact of a given variable on the frailty index, we used an iterative, re-sampling process, similar to "bootstrapping" as detailed elsewhere [19,23]. We performed 1000 iterations where each iteration calculated the baseline and follow up frailty indexes using 80% of their variables, plotted the log of these two frailty indices versus age, and recorded the slope. By analyzing the range of the slopes, we were able to calculate 95% confidence intervals.

To observe the upper limit of the frailty index, the 99^th ^percentiles of each cohort's frailty index was plotted against age. A flattening of this curve (i.e. its approach to a 0 slope) would suggest a common maximum to the frailty index at every age, consistent with earlier observations [24]. Statistical distributions of the frailty index were compared with theoretical models (Goodness of fit by least squares).

Survival analyses were done using bi-variate and multivariate Cox Regression analyses with the frailty index as the independent variable and age and gender as covariates on each of the two survey waves. The survival calculations were based on the available nine year mortality data from the baseline survey. The 18 month follow up survival calculations based from the available seven and a half year (from 18 month interview) mortality data.

### Comparison of the Frailty Index calculated for the PEP Study with earlier estimates

The two calculated indexes, one from baseline and one at follow-up, were compared with previously published indexes, to see how well each fit the following characteristics: 1) The Frailty Index should have a skewed density distribution (histogram) that is well approximated by a gamma distribution [4,8,10] 2) The rate of deficit accumulation (prior estimate is 0.03 per year) [4-6]; 3) The presence of a sub-maximal, age-invariant limit to the Frailty Index (prior estimate is ~0.67) [5,6,8]; and 4) Association of the mean value of the Frailty Index with mortality [4-6,8,10,25].

## Ethics

The study protocol was approved by the Yale Human Investigation Committee, New Haven; all participants provided informed consent at baseline and at follow-up. Ethical approval for secondary analyses was obtained from the Capital District Health Authority, Halifax, Nova Scotia.

## Results

### Construction and characteristics of the Frailty Index at baseline and at follow-up

Of the variables considered, 40 variables that met all frailty index criteria at both baseline and follow-up were chosen (Table 1). Variables were eliminated because they did not meet at least one of the five criteria (unrelated to age and adverse outcome, saturated, or there was already ample representation of the system) or because we had identified 40 variables with which to populate the index. Some potential variables excluded were: Distance walked (up to 20 ft.) (saturated), admitted to hospital in the past year (non-age associated), use of a walking device (sufficient variables (i.e. n = 40) were already included), walking a quarter mile (already accounted for by two variables), measured blood pressure (sitting and standing) (non-age associated), fractures (non-age associated), Parkinson's Disease (low prevalence), amputation (non-age associated), liver disease (not present in both surveys), taking medication (controversial in relation to adverse health outcome), light/medium/heavy sports (Unreliable prevalence and age association), measured vision (saturation), and various tests of physical performance (already accounted for in other variables and 40 variables already populating the index), such as finger tap and turning in a complete circle.

**Table 1:** Health Variables and Cut-points for the Frailty Index

**List of 40 Variables included in the frailty index**	**Cut Point**
Help Bathing	Yes = 1, No = 0
Help Dressing	Yes = 1, No = 0
Help getting in/out of Chair	Yes = 1, No = 0
Help Walking around house	Yes = 1, No = 0
Help Eating	Yes = 1, No = 0
Help Grooming	Yes = 1, No = 0
Help Using Toilet	Yes = 1, No = 0
Help up/down Stairs	Yes = 1, No = 0
Help lifting 10 lbs	Yes = 1, No = 0
Help Shopping	Yes = 1, No = 0
Help with Housework	Yes = 1, No = 0
Help with meal Preparations	Yes = 1, No = 0
Help taking Medication	Yes = 1, No = 0
Help with Finances	Yes = 1, No = 0
Lost more than 10 lbs in last year	Yes = 1, No = 0
Self Rating of Health	Poor = 1, Fair = 0.75, Good = 0.5, V. Good = 0.25, Excellent = 0
How Health has changed in last year	Worse = 1, Better/Same = 0
Stayed in Bed at least half the day due to health (in last month)	Yes = 1, No = 0
Cut down on Usual Activity (in last month)	Yes = 1, No = 0
Walk outside	<3 days = 1, ≤ 3 days = 0
Feel Everything is an Effort	Most of time = 1, Some time = 0.5, Rarely = 0
Feel Depressed	Most of time = 1, Some time = 0.5, Rarely = 0
Feel Happy	Most of time = 0, Some time = 0.5, Rarely = 1
Feel Lonely	Most of time = 1, Some time = 0.5, Rarely = 0
Have Trouble getting going	Most of time = 1, Some time = 0.5, Rarely = 0
High blood pressure	Yes = 1, Suspect = 0.5, No = 0
Heart attack	Yes = 1, Suspect = 0.5, No = 0
CHF	Yes = 1, Suspect = 0.5, No = 0
Stroke	Yes = 1, Suspect = 0.5, No = 0
Cancer	Yes = 1, Suspect = 0.5, No = 0
Diabetes	Yes = 1, Suspect = 0.5, No = 0
Arthritis	Yes = 1, Suspect = 0.5, No = 0
Chronic Lung Disease	Yes = 1, Suspect = 0.5, No = 0
MMSE	<10 = 1, 11–17 = 0.75, 18–20 = 0.5, 20–24 = 0.25, >24 = 0
Peak Flow	See Table 2
Shoulder Strength	See Table 2
BMI	See Table 2
Grip Strength	See Table 2
Usual Pace	See Table 2
Rapid Pace	See Table 2

The list of health deficit variables included in the FI and how they were coded as deficits.

Of the 40 variables included in the Frailty Index, three were continuous, with no clear cut-point for inclusion. These were peak flow, shoulder strength and timed usual pace walk for 20 ft. These variables' deficits were determined by plotting them against the frailty index (without the variables being added) and identifying the value corresponding to 0.2 (Table 2). Of interest, when other continuous variables (grip strength, timed rapid walk of 20 ft.) were plotted against interim frailty indexes, similar cut-points to their published cut offs were found (data not shown).

**Table 2:** Continuous Variable Cut-points

**Variable**	**Deficit for Men**	**Deficit for Women**	**Source of cut point**
Peak Flow (liters/min)	≤ 340	≤ 310	Plotted verses frailty index
Body Mass Index (BMI)	<18.5, ≥ 30 as a deficit. 25-<30 as a 'half deficit'	<18.5, ≥ 30 as a deficit. 25-<30 as a 'half deficit'	Published [34]
Shoulder Strength (kg)	≤ 12	≤ 9	Plotted verses frailty index
Grip Strength (GS in kg)	For BMI ≤ 24, GS ≤ 29 For BMI 24.1–28, GS ≤ 30 For BMI >28, GS ≤ 32	For BMI ≤ 23, GS ≤ 17 For BMI 23.1–26, GS ≤ 17.3 For BMI 26.1–29, GS ≤ 18 For BMI>29, GS ≤ 21	Published [15,22]
Rapid pace Walk (sec)	>10	>10	Published [15]
Usual pace Walk (sec)	>16	>16	Plotted verses frailty index

Deficit cut off values for continuous variables by sex and source of cut off.

**Figure 1:**
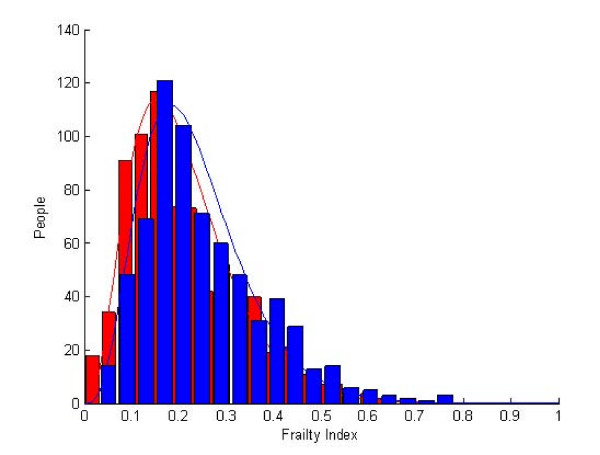
**Frailty Index Distribution**. Gamma distribution fit (lines) of the observed distribution of the frailty index (bar) in the baseline (red) and 18 month follow up (blue) sample.

The baseline and follow up Frailty Index distributions were well correlated to a gamma distribution (Figure 1, r^2^>0.90). At baseline, more people had Frailty Index values between 0–0.15, whereas at follow up, more people had higher Frailty Index values.

**Figure 2:**
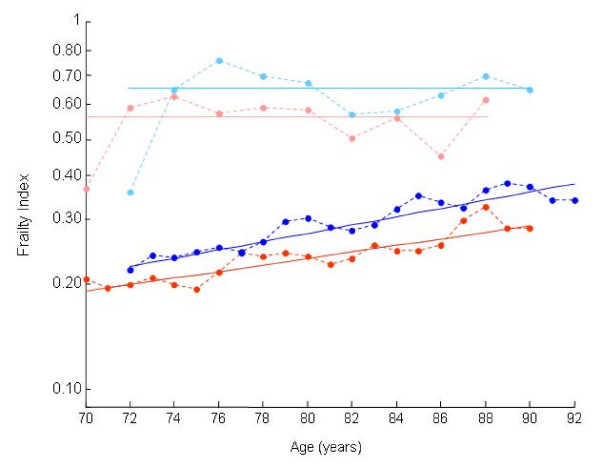
**Frailty Index versus Age Plot**. Frailty index versus age plot of baseline (light and dark red) and 18 month follow up (light and dark blue). Shown here are the average (dark blue/red) and the observed 99^th ^percentile (light blue/red) lines. The slope of the best fit curves shows no accumulation of deficits in the most impaired (99^th^) of the sample. By contrast the follow up average curve has 2.6% deficit accumulation per year. The baseline average curve has a 2.0% deficit accumulation per year; the 99^th ^percentile slope also shows no accumulation of deficits with age.

In relation to age, the baseline average slope of the deficit accumulation line was 0.020 (95% confidence interval 0.014–0.026); i.e. on average, the estimated mean rate of deficit accumulation was 0.020 per year (Figure 2). For the cohort at follow-up, the slope of the line relating deficits to age was 0.026 (95% confidence interval 0.020–0.032).

In investigating the upper limits (99% sample) to the Frailty Index, we noted that both the baseline and follow-up cohorts no longer showed a relationship between age and deficit accumulation (Figure 2). Indeed, the best fit line of the 99% sample has a slope statistically indistinguishable from 0. The upper limit using the baseline cohort was around 0.6, while the limit using the follow up cohort was about 0.7 (there were four individuals with slightly higher frailty values).

**Figure 3:**
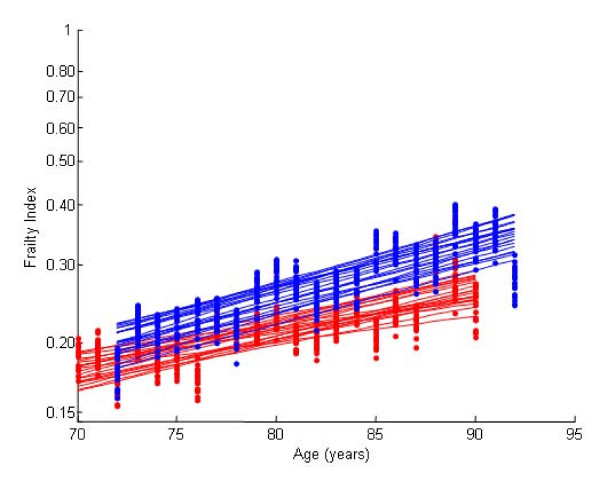
**Variance in the Slope of the Frailty Index**. The Bootstrapping of the frailty index. The frailty index was created and plotted 1000 times, each time randomly picking 80% of the variables of the index. Twenty iterations are shown here. The experimental and best fit regression lines of the average index values are shown in the baseline (red) and follow up (blue).

In both cohorts, the construction of the Frailty Index showed little sensitivity to which variables were included (Figure 3). The differences in slopes were negligible when 80% of the variables were re-sampled; differences in the intercepts of the relationship between age and deficit accumulation were more evident, but within non-overlapping confidence intervals (Figure 3).

### Mortality in relation to the Frailty Index

The baseline and follow up cohort's Frailty Indexes were each associated with mortality. In the bivariate Cox regression analysis, sex, age and the frailty index were each significant predictors of survival in the baseline and follow up cohort (Table 3). In the multivariable analysis, all these variables were significantly related to mortality at both baseline and at follow up.

**Table 3:** Cox Analyses

		**Cox Analyses**			
		**Baseline**		**Follow up**	
Analysis	Variable	HR	95% CI	HR	95% CI
Bi-variate	Age	1.09	1.07 – 1.11	1.09	1.06 – 1.11
	Frailty Index	1.03	1.02 – 1.04	1.05	1.04 – 1.05
	Male Sex	1.46	1.16 – 1.82	1.37	1.07 – 1.74
Multi-variate^a^	Age	1.08	1.06 – 1.10	1.06	1.04 – 1.09
	Frailty Index	1.03	1.02 – 1.04	1.04	1.04 – 1.05
	Male Sex	1.80	1.42 – 2.27	1.71	1.33 – 2.20

Cox Regression analyses of the baseline and follow up. Calculations were based on monthly follow ups for nine years from the baseline interview (or seven and a half years from the 18 month follow up). The frailty index hazard ratios (HR) are calculated with % levels of the index (i.e. the HR measures a change of 0.01 on the index).

^a ^Analysis done with Age, Sex and Frailty Index as covariates

## Discussion

In a secondary analysis of the Yale Precipitating Events Project, a Frailty Index was constructed for a baseline and a follow-up cohort, respectively. Each step in the process was described, to allow a precise account of what constitutes a health deficit for this purpose, how to select which health deficits to include in a frailty index, how to operationalize any possible deficit (ordinal, continuous and binary) to a range of 0–1 and which characteristics of the frailty index (nature of the distribution; slope in relation to age; presence of a limit) seem to be broadly replicable. Several reproducible characteristics (e.g. the distribution, the slope and limit of deficit accumulation) of each Frailty Index were provided so that they maybe used, as in previous papers [4-11], to describe the overall frailty state of the group. The baseline Frailty Index showed a rate of accumulation to be 0.020 per year (per 1 year increase in age) with an upper limit to the frailty index of about 0.60 while the follow up showed a rate of 0.026 deficits accumulated per year with a limit around 0.70 (Figure 2).

We used a re-sampling by variable procedure to construct confidence intervals for the slopes of the lines (Figure 3). This procedure gives us information about the frailty construct, showing that a range of deficits can in fact be combined to give a result that is informative in the aggregate. In other words, the slope depends on the overall behaviour of the deficit accumulation, and is not driven by a small number of variables. In this regard, earlier work has shown reasonable consistency of the rate of deficit accumulation across community-dwelling random samples [6]. Here, we noted that the follow up cohort had frailty index characteristics – frailty index values, rate and limit similar to those of previously studied community dwelling random samples. Most notable is the 0.03 accumulation of deficits and the age independent limit to frailty of 0.67. The baseline sample had lower estimates – a lower average Frailty Index and a lower maximal limit. This suggests that the baseline cohort was not as frail as the follow up cohort.

The relationship between the Frailty Index and mortality is of interest on several grounds, but here is presented chiefly because it represents a relevant and non-arbitrary test of predictive validity. This is important because predictive validity is one of two types of so-called criterion validation, the other being validation against a so-called "gold standard" [21]. As there is no gold standard for frailty assessment, predictive validation is an important method of validating any approach to frailty operationalization. Note that our intent in checking the ability of the frailty index to predict mortality is validation of the index, rather than developing a mortality prediction index that included frailty. If the frailty index were meant to be a mortality prediction instrument, there might be a rationale for weighting several items, particularly age [26]. One notable result from the Cox analyses is that including the Frailty Index increased the impact of being male on mortality. This likely reflects the observation from earlier studies that while men accumulate fewer deficits than do women, any given level of deficit accumulation is more lethal for them and at any given age, females seem to be more frail than males [6,11].

The relationship with mortality is also important in understanding how deficit accumulation might operate. Classically, Gompertz described the rate of mortality being exponentially related to age [27]. Equally unsurprisingly, mortality exponentially increases with the accumulation of deficits [5,8,19]. In addition, acceleration of deficit accumulation is characteristic of older people prior to death [8].

Our data must be interpreted with caution. Not all items had established cut-points. In addition, cut-points can be difficult to apply across a sample that covers many ages, as the effects of continuous traits can be age-specific.[28] Our approach derived cut-points based on the "interim frailty index" procedure described above. In addition, the sample is small, so that any individual estimates can be unstable; this is where aggregation of items in a frailty index can be helpful, and where the re-sampling strategy is useful.

Our paper also has some strengths. In replicating many of the characteristics of a frailty index in a new sample, we can give additional assurance of the robustness of the approach. By spelling out in detail how each step in constructing a frailty index can be undertaken, and by submitting to an open access journal, we are aiming to make the method widely available. We have also made more precise a method for establishing cut-points for variables that were not constructed for inclusion in a frailty index, thereby further allowing the method to be used. In this regard, the relationship of any given variable to a mean frailty index score of 0.2 might seem arbitrary. In a study that related the frailty index approach to the phenotypic definition of frailty popularized from the Cardiovascular Health Study [22], 0.2 corresponded to the mean frailty index value for persons defined as "pre-frail" [7,22]. A more recent paper from another group used the 0.2 cut-point on a so-called "deficit index" to distinguish people who were "robust" form those who were "pre-frail"[29]. Finally, like many health surveys, the PEP study has many more variables than are needed to construct a 40-item frailty index. Several eligible variables were not included only because we had reached our target of a 40-item Frailty Index. There is no scientific reason not to include more – we have constructed an frailty index of 70 items. On the other hand, a recurring concern about the frailty index has been the feasibility of calculating it if a lot of variables are used [30]. Here, as in some earlier studies, [7,19,31] we have selected variables at random (boot-strapping) from a list of eligible variables to make up the Frailty Index and have again shown that the results are insensitive to the precise composition of the index.

Change in the health status of elderly people is an obvious concern to clinicians and to population planners. In the next round of analyses, we will be interested to know whether the changes in the frailty states (baseline frailty state versus the follow up state) can be described using a so called "stochastic" transition model [32] which we have evaluated in other community-dwelling elderly samples, although not with ones that include as many performance measures as the PEP study [33]. This intriguing possibility is motivating further inquiries by our group.

## Conclusion

A systematic process for creating a Frailty Index was presented for the Yale Precipitating Events Project, a well studied cohort in which deficit accumulation previously had not been evaluated. The process allows operationalization of the frailty index to be carried out in other datasets. The frailty index reveals how frailty, understood as a vulnerability state with an increased risk of adverse outcomes, can be quantified. This method of quantifying frailty can aid our understanding of health and frailty-related health characteristics and outcomes in older adults.

## Competing interests

No sponsor had a role in the decision to undertake these analyses or to submit the study for publication. Each author asserts no proprietary interest in the result and no financial conflict of interest.

## Authors' contributions

Sam Searle carried out the analyses and wrote the first draft as part of his PhD program. Arnold Mitnitski supervised these analyses. Evelyne Gahbauer provided the datasets. Thomas Gill is the PI of the PEP study and critiqued each draft. Kenneth Rockwood conceived of the idea with Arnold Mitnitski, with whom he arranged funding and co-wrote the first and subsequent drafts. All authors reviewed and approved the final draft of the paper.

## Pre-publication history

The pre-publication history for this paper can be accessed here:


